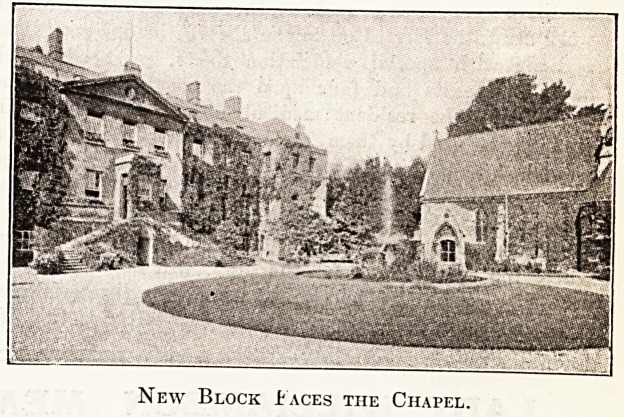# The Extension of Radcliffe Infirmary, Oxford

**Published:** 1913-08-23

**Authors:** 


### THE EXTENSION OF RADCLIFFE INFIRMARY, OXFORD.

### A Tour of the New Block with Captain G. C. Rynd, Secretary.

The visit of the members of the British Hospitals Association to Oxford in June gained added interest from the fact that the visitors were invited to inspect the new out-patient unit which is in process of completion at the Radcliffe Infirmary and Oxford County Hospital. To the experienced hospital manager an uncompleted extension scheme offers as much as, if not more, valuable experience than an inspection of a completed undertaking. For the management of such a transition is itself an interesting thing, and in the still exposed mechanism of construction the design and quality of pipes, mains, and countless other matters can be examined better perhaps than when a trade finish has been added. Captain G. C. Rynd, the secretary and superintendent, courteously explained to our Commissioner the scope of the extension scheme.

"The Radcliffe Infirmary," he began, "like every other institution in Oxford, has associations; and perhaps in no other institution has a fact of this kind conditioned to the same extent, and in the end successfully, any modern hospital. The original institution, which was founded in 1770, is a Georgian building, with the charm and unpretentious dignity of that period of architecture. The fact that we have been able to preserve this untouched, while at the same time satisfying the demands of modern hospital construction, is one of the first points of interest in the institution. The old building, with the large separated chapel at right angles to it on the north side of the entrance gates, already formed two sides of the characteristic University quadrangle. The new outpatient unit, which we are now completing, gives us the third side, and when it is finished the quadrangle will be properly squared, with buildings on three sides and railings and gates upon the fourth."

"The original building has been left untouched?"

"Untouched as to its construction, but changed as to its use. It is now the administration block. The old wards, which ran parallel to the front, and consequently had no cross-ventilation, have been divided into nurses' bedrooms, the size of which is, without exaggeration, quite double that given in usual up-to-date nurses' homes. Above, again, are the cubicles for the domestic staff; and now that the nursing staff has been increased as new wards have been built, we have bought in turn the two houses opposite to the hospital on the other side of the Woodstock Road, which have made an admirable nurses' home, and the purchase of which, at a cost of £1,500, has involved us in far less expense than that of a new block. The wards, which are comprised in four blocks, contain some 155 beds. Three of them, together with the theatre unit, open out of a long corridor, and all have been added at different periods. So when the new buildings are finished the institution will represent in miniature the evolution of hospital design in its individual blocks, which encroach upon the characteristic Oxford garden that once formed the back of the original house. The Briscoe Ward block was built in 1863, the women's block in 1871, and the children's block in 1877; these two last were respectively enlarged fifteen years after they were built. The men's block of two wards was built in 1894, the operating theatre in 1899, and the new outpatient unit, we may say, before the present year is out."

"What does the new block consist of?"

"Roughly speaking, of an out-patient unit and of special departments. The building has a basement, ground, first, and second floors. Let us visit these in turn, as far as their present state allows."

On the way to the basement Captain Rynd explained that building began in September 1911, and that it was hoped to have finished by June. It is now probable that the department will be ready for occupation at the end of the Long Vacation. Making our way downstairs between the workmen and their tools, the basement was seen to consist of various store-rooms, including those for drugs and bandages, a mortuary, with cold storage and an electric lift directly connecting this with the pathological laboratory on the second floor, air ducts, and a main duct for electric, gas, and water mains. "Wherever you go throughout the new building," he continued, "you will notice that pipes are visible. Our Hon. Staff were very emphatic on the need for having them accessible at every possible part of their course: accessibility is all the more important, because it takes only about five years for any pipe in Oxford to become furred owing to the nature of the water." Upstairs the

New Block Faces the Chapel.

centre of the ground floor is occupied by the out-patient hall, which measures 81 by 26 feet, round which are grouped six consulting rooms, an almoner's room, a waiting hall, with patients' entrance and lavatories ventilated by electric exhaust fans on either side of it, a porter's room adjoining the staff and students' entrance, and the mortuary chapel, given by Mr. Randall Higgins, which is over the mortuary, next to which the lift, already mentioned, ascends. Also on the ground floor and at the inner end of the new wing is the casualty block, with its own entrance and separate heating system. The casualty block consists of a dressing room, casualty room, operating-room entrance, and two recovery rooms on one side, and the dispensary waiting hall on the other. The centre of the floor is occupied by a corridor and the dispensary proper. Another electric lift, large enough to take a patient in bed, connects the ground floor with the upper floors of the new block.

"The floors, generally speaking, also the dados on staircases and corridors," said Captain Rynd, "are of terrazzo divided by lead lines into squares to prevent cracks. In the operating room, I may also mention, lighting is to include Zeiss lamps. If we go up now to the first floor you will be able to see how the special departments are grouped. The first noticeable feature of the first floor is the asphalt flat, which forms the roof of the consulting rooms on the north side of the out-patient hall. At the back of this flat rises the glass roof of the hall itself. Standing on the flat with your back to the chapel, which forms the north side of the quadrangle, facing the glass roof of the out-patient hall, on your left is the resident medical officers' flat, opposite are the therapeutic *x*-ray room, the photographic *x*-ray room, a dark room; and on your right are the ear, nose, and throat and electro-therapeutic rooms, with the dental workshop. As it is not expected that the ear, nose, and throat room will be constantly required, it is hoped to carry out massage treatment in the intervals, and to prevent confusion and to give a clear space for such work, examination cupboards have been fixed to the walls, which, when not required for nose and throat work, will be shut, like wardrobes, leaving ample free space in the centre of the room. The windows in the corridor behind the *x*-ray rooms have two details of interest. The sash-cords are Hookham's patent, steel core covered with copper wire, which run easily and last well. The windows can all be cleaned from the inside, and are made on a hinge which enables the frame to be swung into a horizontal position. While we are on this floor you will see that there is another flat over the casualty department, but the space there is largely taken up with ventilation shafts. Our aim has been to ventilate every room, such as the dark rooms, in which work has to be carried on in the absence of ordinary light and air with special ventilation ducts, most of which have their exits and fans upon the casualty roof. The flat outside the resident medical officers' quarters will probably be used by them, at least in warm weather, as a kind of open-air sitting-room. The second and top floors form practically our bacteriological and pathological department. Over the resident medical officers' flat are the professors' room, the registrar's room, and library, where the examination for the M.B. will be held. Over the *x*-ray rooms are the clinical laboratory (bacteriology) and the *post-mortem* room. Beside the latter is the lift which, as you saw when we were in the basement, communicates at its lower level with the mortuary. The space over the electro-therapeutic department is occupied by a lecture room. The bacteriological department has been designed in consultation with Professor Dreyer, Professor of Pathology in the University. The above is a rough outline of the main features of the extension, which consists of a basement, out-patient and casualty units on the ground floor, a resident medical officers' flat and special departments on the first floor, and a pathological unit on the second. The plans, of course, must be studied to appreciate the way in which the departments have been designed and the nature of the site."

"What is the cost of the extension?"

"The estimate is £25,000, of which we have raised some £8,000 at the present time. We hope also that the new facilities which the extension will give for teaching purposes may lead to our receiving a grant from the University. At present we receive only grants from the various Colleges, while I understand that Addenbrooke's Hospital, Cambridge, is given £300 a year from the sister University. We are very pleased with the style of the building which Mr. Edward Warren, F.S.A., F.R.I.B.A., has designed. It is simple but dignified, and harmonises well with the old Georgian administrative block that is the nucleus of the present, and was once the original, hospital. By the way, there are two points of detail that may be of interest. Some years ago we built balconies to all the wards. These are now being enlarged, so that they will allow of any one of the beds being wheeled past another, which was not possible when the balconies were first built. The second matter concerns the interior of the ward. In order to prevent the heads of the beds from damaging the walls, the point at which these meet the floors, instead of having the usual concave corner, is filled up with solid concrete on expanded metal. This forms a rounded convex surface, which collects no dust, can be cleaned in a moment, and prevents the head of the bed from touching the wall."

## Figures and Tables

**Figure f1:**